# 591. Comparison of Fluconazole to Other Antifungal Agents in Allogeneic Hematopoietic Cell Transplant Recipients: A Meta-Analysis of Randomized Clinical Trials

**DOI:** 10.1093/ofid/ofab466.789

**Published:** 2021-12-04

**Authors:** Ce Cheng, Iloabueke Chineke, Ali McBride, Alejandro Recio-Boiles, Lakshmi Saritha Ainapurapu, Chenyu Sun, Niharika Vennelaganti

**Affiliations:** 1 University of Arizona College of Medicine at South Campus , Tucson, Arizona; 2 The University of Arizona Cancer Center, Tucson, Arizona; 3 The University of Arizona College of Medicine/Banner University Medical Center South, Tucson, Arizona; 4 AMITA Health Saint Joseph Hospital Chicago, Chicago, Illinois

## Abstract

**Background:**

Invasive fungal infections (IFI) are adverse complications of allogeneic and autologous hematopoietic stem cell transplantation (HSCT) with significant mortality and morbidity. Randomized Controlled Trials (RCT) have addressed the optimal anti-fungal prophylaxis regimen. However, the consensus for an anti-fungal prophylaxis regimen has remained elusive. Hence, we performed a meta-analysis of currently available RCTs comparing the efficacy of fluconazole vs. other antifungal agents including voriconazole, micafungin, and itraconazole in the endpoint of preventing IFI.

**Methods:**

Randomized controlled trials were retrieved from PubMed, according to our inclusion criteria. The relative risk (RR), hazard risk (HR), and 95% confidence intervals (CI) were calculated. A random effect or fixed-effect model was used to calculate the pooled HR, based on heterogeneity. All statistical analyses were performed using RevMan software and R Core Team, and all p-values were two-tailed, and the significance level was 0.05.

**Results:**

Ten RCTs were selected involving 2654 pts. Our results showed fluconazole is statistically inferior to other agents that include voriconazole, micafungin, and itraconazole with regards to the endpoint of a lower incidence of IFI (RR: 1.05; 95%CI: 1.02, 1.08; p=0.0002, I^2^=5%). However, subgroup analysis showed no statistical difference between fluconazole vs. other agents to prevent breakthrough proven IFI (HR: 0.76; 95%CI: 0.47, 1.23; p=0.27, I^2^=0%). Our subgroup analysis further showed that other agent’s group might have a superior role in preventing aspergillus compared with fluconazole (HR: 0.64; 95%CI: 0.44, 0.94; p=0.02, I^2^=0%), but no significant advantages over fluconazole for candidiasis (HR: 0.96; 95%CI: 0.45, 2.07; p=0.92, I^2^=0%).

Successful Rate Without Incidence of IFI

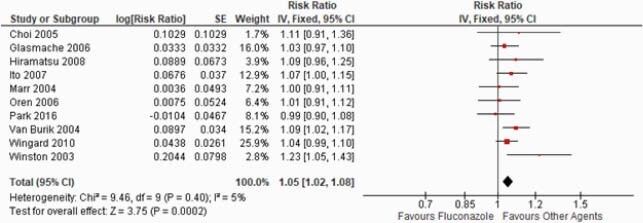

Figure 1. Successful Rate Without Incidence of IFI

Proven IFI vs. Suspected IFI

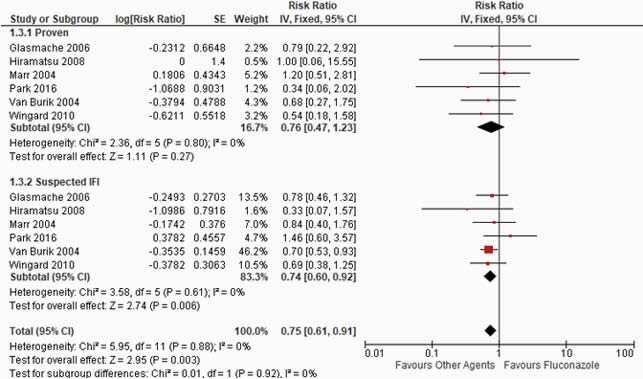

Figure 2. Proven IFI vs. Suspected IFI

Candidiasis vs. Aspergillus

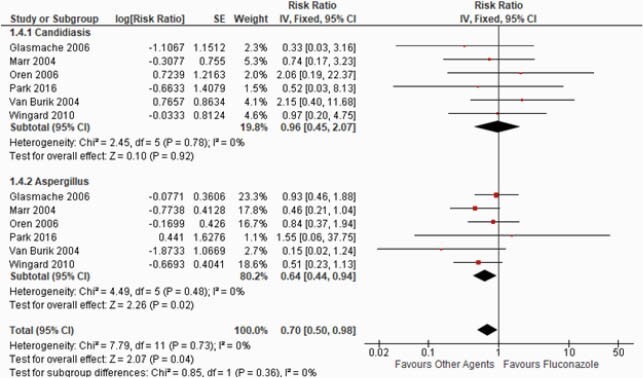

Figure 3. Candidiasis vs. Aspergillus

**Conclusion:**

This meta-analysis yield data that suggests fluconazole might be inferior to other agents in preventing IFI in all intent to treat patients undergoing HSCT. However, fluconazole is non-inferior in preventing proven IFI and candidiasis IFI based on our results. Thus, we continue to recommend fluconazole in selected patients who require anti-fungal prophylaxis. More RCTs are needed in the future to demonstrate the drug of choice for anti-fungal prophylaxis and address patient selection characteristics.

**Disclosures:**

**All Authors**: No reported disclosures

